# Xenopericardial roll graft replacement for infectious pseudoaneurysms and graft infections of the aorta

**DOI:** 10.1186/s13019-015-0343-5

**Published:** 2015-10-27

**Authors:** Hiroshi Kubota, Hidehito Endo, Mio Noma, Hikaru Ishii, Hiroshi Tsuchiya, Akihiro Yoshimoto, Yu Takahashi, Yusuke Inaba, Yoshifumi Nishino, Masao Nunokawa, Yutaka Hosoi, Tooru Ikezoe, Masaru Nemoto, Yoshihisa Makino, Yoko Nemoto, Mitsuru Matsukura, Masanori Sugiyama, Nobutsugu Abe, Hirohisa Takeuchi, Gen Nagao, Eri Kondo, Osamu Yanagida, Hideaki Yoshino, Kenichi Sudo

**Affiliations:** 1 Department of Cardiovascular Surgery, Kyorin University, 6-20-2, Shinkawa, Mitaka, Tokyo 181-8611 Japan; 2 Department of Vascular Surgery, Tokyo University, Tokyo, Japan; 3 Department of Gastroenterological Surgery, Kyorin University, Tokyo, Japan; 4 Kosei General Hospital affiliated to Rissho Kosei-Kai, Tokyo, Japan; 5 Department of Cardiology, Kyorin University, Tokyo, Japan; 6 Jiseikai Nomura Hospital, Tokyo, Japan

**Keywords:** Aortic operation, Pseudoaneurysm, Biomaterials, Esophageal injury, Esophageal perforation, Infection, Graft infection, Xenograft, Equine pericardium, Bovine pericardium

## Abstract

**Background:**

Which graft material is the optimal graft material for the treatment of infected aortic aneurysms and aortic graft infections is still a matter of controversy. Orthotopic aortic reconstruction with intraoperatively prepared xenopericardial roll grafts without omentopexy was performed as the “initial” operation to treat aortic infection or as a “rescue” operation to treat graft infection. Mid-term outcomes were evaluated.

**Methods:**

Between 2009 and 2013, orthotopic xenopericardial roll graft replacement was performed to treat eight patients (male/female: 6/2; mean age: 69.5 [55–80] yr). Graft material: equine/bovine pericardium: 2/6; type of operation: initial 4/rescue 4; omentopexy 0. Additional operation: esophagectomy 2. Mean follow-up period: 2.6 ± 1.6 (1.1–5.1) years.

**Results:**

Replacement: ascending 3, arch 1 (reconstruction of neck vessels with small xenopericardial roll grafts), descending 3, and thoracoabdominal 1. Pathogens: MRSA 2, MSSA 1, Candida 1, E. coli 1, oral bacillus 1, and culture negative 2. Postoperative local recurrence of infection: 0. Graft-related complications: stenosis 0, calcification 0, non-infectious pseudoaneurysm of anastomosis 2 (surgical repair: 1/TEVAR 1). In-hospital mortality: 2 (MOF: initial 1/rescue 1); Survival rate exclusive of in-hospital deaths (~3 y): 100 %, but one patient died of lung cancer (3.6 yr).

**Conclusions:**

Because xenopericardial roll grafts are not composed of synthetic material, the replacement procedure is simpler and less invasive than the standard procedure. Based on the favorable results obtained, this procedure may have the possibility to serve as an option for the treatment of aortic infections and aortic graft infections not only as a “rescue” treatment but as an “initial” treatment as well.

**Electronic supplementary material:**

The online version of this article (doi:10.1186/s13019-015-0343-5) contains supplementary material, which is available to authorized users.

## Background

The standard procedure for treating infected aortic aneurysms and aortic graft infection after aortic procedures is resection of the infected aorta/graft, debridement of the surrounding tissue, in situ graft replacement. Rifampicin-soaked Dacron grafts, cryopreserved arterial homografts are clinically accepted. The omentopexy is the most reliable barrier to the infection. However, for a variety of reasons, we often face the patients whose omentum cannot be used. Xenopericardial roll graft replacement has been performed to treat native aorta/graft infections without omentopexy.

## Methods

Between 2009 and 2013, orthotopic xenopericardial roll graft replacement was performed to treat eight patients, six male and two female; mean age of 69.5 (55–80) years old (Table 1). Four patients had a past history of aortic surgery with a synthetic graft (Cases 4, 5, 7, 8). In the other four patients an infected aorta was replaced by a xenopericardial roll graft as an initial operation (Cases 1, 2, 3, 6). Three of the four patients had a history of abdominal surgery. The other patient (Case 3) showed sepsis and multiple organ failure due to the deep sternal wound infection (DSWI).

**Table 1:** Patient profiles

Case No.	Age (yr)	Previous aortic operation	Site and type of operation		Aortic pathology	Previous operation	Source of infection	Organism	Pericardium used	Omento-pexy
1	75	-	Ascending	Initial	Pseudoaneurysm	Subtotal gastrectomy + omentectomy	Unclear	MSSA	Equine	-
2	79	-	Arch	Initial	Pseudoaneurysm	Y-graft	Unknown	Negative	Equine	-
3	63	-	Thoraco-abdominal	Initial	Dissection	Arch	DSWI	MRSA	Bovine	-
4	72	Descending	Descending (+esophagectomy + jejunostomy)	Rescue	True aneurysm	-	Esophageal perforation	Candida albicans E. coli, Streptococcus anginosus	Bovine	-
5	55	Ascending	Ascending	Rescue	Dissection	-	Unknown	Negative	Bovine	-
6	80	-	Descending	Initial	Pseudoaneurysm	Liver abscess drainage	Liver abscess	E. coli	Bovine	-
7	59	Ascending	Ascending	Rescue	Dissection	-	DSWI	MRSA	Bovine	-
8	73	Descending + rescue TEVAR	Descending (+esophagectomy)	Rescue	True aneurysm	-	Esophageal perforation	Oral bacillus	Bovine	-

*TEVAR* thoracic endovascular aortic repair, *DSWI* deep sternal wound infection, *MSSA* methicillin-sensitive Staphylococcus aureus*, MRSA methicillin-resistant Staphylococcus aureus*

The source of the causative organism was a DSWI in two patients and a liver abscess in one patient. In other two patients, the left pleural cavity had become contaminated as a result of esophageal perforation. Causative organisms were detected in six (75 %) patients. Methicillin resistant staphylococcus aureus (MRSA) and Escherichia coli were detected in two patients, and methicillin-sensitive Staphylococcus aureus, Candida albicans, Candida glabrata, Streptococcus anginosus, and an oral bacillus were detected in one patient each. Simple esophagectomy, esophagectomy with cervical esophagostomy and jejunpstomy were performed in one patient each.

**Fig. 1:**
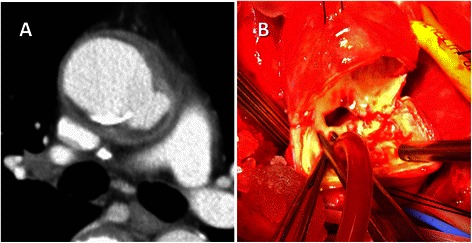
**a** Computed tomography showed a pseudoaneurysm and abscess in the atherosclerotic lesion of the ascending aorta (Case 1). **b** There was a large purulent pericardial effusion. The abscess extended to just above the main trunk of the left coronary artery, and the aorta was “punched out”. The cultures of both pericardial effusion and resected aorta were positive for methicillin-sensitive *Staphyrococcus aureus*.”

Aortic infection and aortic graft infection were diagnosed on the basis of blood cultures, the leukocyte count, serum C-reactive protein level, serum procalcitonin level, and clinical findings taken together. Pericardial effusion, perigraft air and abscess formation, and rapid growth of the “punched out” area were important local image signs of aorta/graft infection (Fig. 1a, b).

Written informed consent was obtained from all patients before the procedure.

We followed the ‘Prevention and Treatment of Infective Endocarditis (Japanese Circulation Society 2008)’ guidelines in regard to the antibiotic regimen. In accordance with the guidelines for active infective ‘native’ valve endocarditis, the infection control team of Kyorin University administered the most appropriate antibiotic treatment to the patients in whom the causative organism had been identified. In culture-negative situations, we administered empiric antibiotic treatment. When inflammatory marker values had become normal and the diagnostic images no longer showed evidence of the infection, antibiotic treatment was stopped until 4 to 6 weeks. Otherwise, antibiotic treatment was continued.

### Surgical procedures

**Fig. 2:**
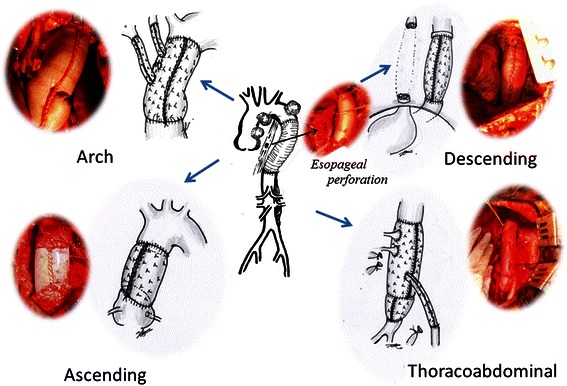
Xenopericardial roll graft replacement of the aorta

Four patients underwent replacement of the ascending aorta or aortic arch with an open distal anastomosis during deep hypothermic circulatory arrest with intermittent-pressure-augmented retrograde cerebral perfusion [1–5]. The myocardium was protected by intermittent retrograde infusion of cold blood cardioplegia solution followed by continuous cold blood perfusion. In one patient, because of the postoperative dense adhesions after treating DSWI, the heart was not exposed at all (Case 7). Three patients underwent replacement of the thoracic descending or thoracoabdominal aorta with an open proximal anastomosis during deep hypothermic circulatory arrest. In one patient, (Case 4) because there was a clampable segment of the aorta, replacement of the descending thoracic aorta was performed under normothermic non-infected beating heart with lower body circulatory assistance (Fig. 2).

#### Reconstruction of the ascending aorta

**Fig. 3:**
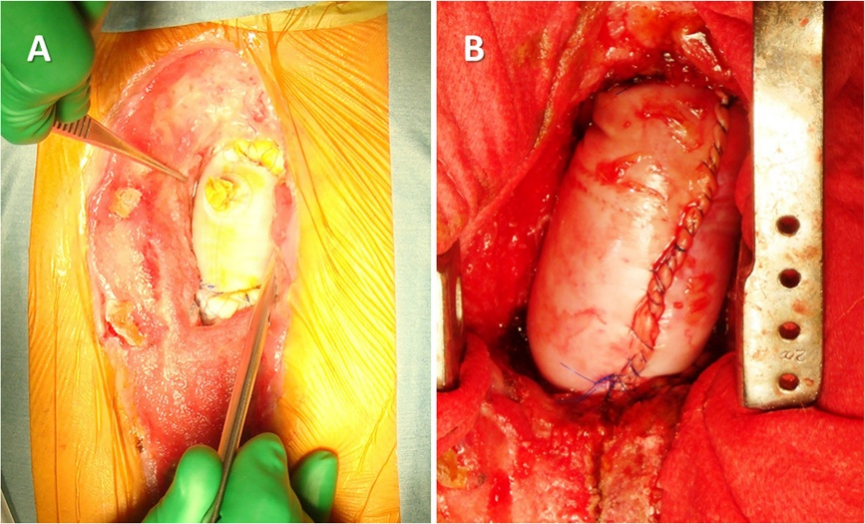
**a** Deep sternal wound infection after ascending aortic replacement (Case 7). After radical debridement of the sternum followed by months of vacuum-assisted wound closure, the wound was well granulated. **b** Bovine pericardial roll graft replacement was performed. The heart was not exposed, and simple deep hypothermic circulatory arrest was used.

The inflow route of the cardiopulmonary bypass was inserted via the femoral artery. A 10 cm × 10 cm unbranched xenopericardial sheet (XGA-400; Edwards Lifesciences, Irvine, CA, USA) was used [6]. It was sutured to the posterior side of the transected aorta and rolled up by continuous suturing with 4–0 polypropylene. Because xenopericardium exhibits only weak anisotropic properties with increasing strain, the pericardial margin to start suturing was selected without considering the orientation [7]. When the corners of the pericardial sheet met, the suture was tied, and the same thread was used to stitch the two sides of the pericardial sheet continuously to form a cylinder. The graft was then clamped, the cardiopulmonary bypass was resumed, and the patient was warmed. The proximal ascending aorta was transected, the pericardial sheet was trimmed, and an anastomosis was created (Fig. 3a, b, Additional file 1: Video S1).

Additional file 1:
**Replacemenet of the ascendingaorta.** (WMV 9844 kb)

#### Reconstruction of the aortic arch

**Fig. 4:**
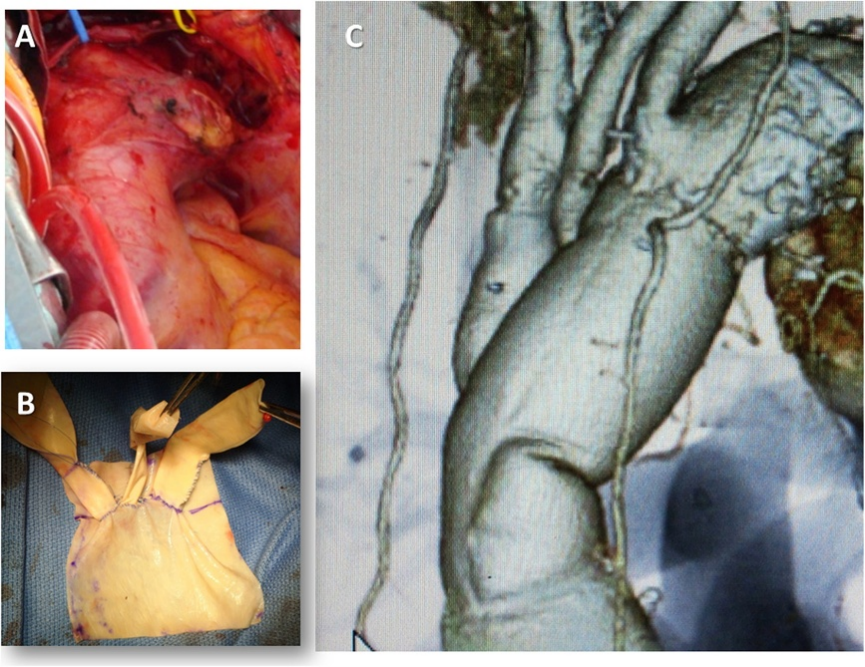
**a** The aneurysm was located in the proximal aortic arch (Case 2). **b** Three-branched pericardial sheet. **c** Postoperative 3-D computed tomography revealed no stenosis or dilatation of the xenopericardial roll graft branches

Three holes in a row, two 10-mm holes and one15-mm hole, were made 5 mm apart in a 10 cm × 10 cm equine pericardial sheet so that the last hole [8]. Then three rectangular pericardial sheets were cut from another pericardial sheet, and each of them was sutured to the circumference of a hole and formed into a cylinder by continuous suturing with 5–0 polypropylene. To determine the optimal length of the graft to prevent kinking of the branches, the sutures placed to form the branches were stopped 20–30 mm from origin of the branch, and the rest was left open (Fig. 4b). Because the infected pseudoaneurysm was located in the proximal portion of the arch, the aorta was transected between the left subclavian artery and the left common carotid artery (Fig. 4a). One side of the original pericardial sheet was anastomosed. The third branch was cut to obtain the proper length and anastomosed to the left common carotid artery. The second branch was used as an inflow route for the cardiopulmonary bypass. The graft was clamped, and antegrade perfusion was restored via the branch. While warming the patient, the proximal branch was anastomosed to the brachiocephalic artery. (Reconstruction of the ascending aorta. 4b, c, Additional file 2: Video S2).

Additional file 2:
**Replacement of the aortic arch.** (WMV 10647 kb)

#### Reconstruction of the thoracic descending aorta and thoracoabdominal aorta

**Fig. 5:**
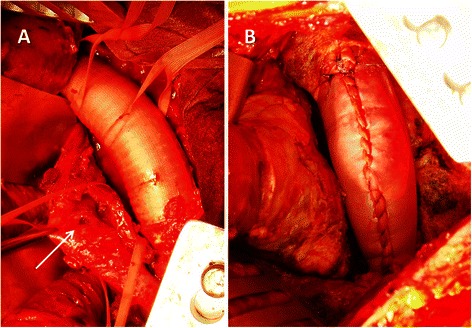
**a** Esophageal perforation (arrow) after replacement of the descending thoracic aorta (Case 4). **b** A xenopericardial roll graft replacement. The proximal anastomosis was reinforced with a xenopericardial strip.

A 10-mm hole was created, and a 3.5 × 10 cm rectangular pericardial sheet cut from another 10 × 10 cm pericardial sheet was sutured to the circumference of the hole in the same fashion. After completing proximal anastomosis, the roll graft was clamped, and antegrade perfusion was restored via the branch. While warming the patient, the distal anastomosis was performed. One patient (Case 6) underwent simple replacement of the infected aorta; one patient (Case 4) underwent esophagectomy and creation of a cervical esophagostomy and jejunostomy concomitant with replacement of an infected Dacron graft (Fig. 5); and one patient (Case 8) underwent simple esophagectomy concomitant with replacement of an infected Dacron graft. To reconstruct the thoracoabdominal aorta (Case 3.), the iliac arteries were reconstructed by using other xenopericardial sheets.

## Results

The mean follow-up period was 2.6 ± 1.6 (1.1–5.1) years. No patients showed any evidence of a postoperative thromboembolic event. Antibiotic treatment could be ceased within 10 weeks in all patients. The patient in case 3, who had multiple organ failure (MOF) secondary to DSWI preoperatively, died on postoperative day (POD) 8. In case 8, it took a long time to dissect the adhesion of the pleural cavity. A long cardiopulmonary bypass time and deep hypothermia time caused excessive bleeding after ceasing the cardiopulmonary bypass and difficult hemostasis and led to early death (POD 1). The other five patients are alive and well.

**Fig. 6:**
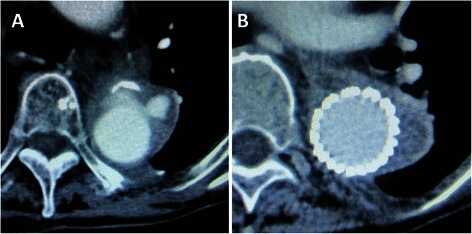
**a**, **b** TEVAR to treat the pseudoaneurysms (Case 4) Case 4 required TEVAR to treat pseudoaneurysms of the anastomoses and to treat pseudoaneurysms of the suture line between the two edges of the pericardial sheet

One patient (Case 4) underwent esophageal reconstruction with a stomach roll 3 months postoperatively. One patient (Case 2) died of lung cancer 45 months postoperatively. All patients who survived have been followed up by means of regular computed tomography examinations, but none of the patients showed evidence of local recurrence of the infection or graft stenosis, calcification, or dilatation, including of the branches. One patient (Case 4) required thoracic endovascular aortic repair (TEVAR) to treat pseudoaneurysms of the anastomoses and the suture line between the two edges of the pericardial sheet (Fig. 6). Another patient (Case 7) required emergency surgery to stop sudden bleeding from the non-infected proximal anastomosis three weeks postoperatively, and intraoperative exploration confirmed a coarse, loose suture. Neither patient showed any signs or symptoms of infection. A total of three patients died, and the other five patients are well and alive during mid- to long-term follow-up (Table 2).

**Table 2:** Postoperative course

Case No.	Site of operation		Follow up (mo.)	Local recurrence	In-hosp. death	Postoperative additional procedures	Results
1	Ascending	Initial	62	-	No		Alive
2	Arch	Initial	45 (lung Ca.)	-	No		Death
3	Thoraco-abdominal	Initial	8POD (MOF)	-	Yes		Death
4	Descending (+esophagectomy)	Rescue	27	-	No	1. Esophageal reconstruction	Alive
						2. TEVAR	
5	Ascending	Rescue	26	-	No		Alive
6	Descending	Initial	14	-	No		Alive
7	Ascending	Rescue	13	-	No	1.Hemostasis	Alive
						2. Pectoralis major muscle plombage	
8	Descending (+esophagectomy)	Rescue	1POD (MOF)		Yes		Death

## Discussion

Complete removal of the infected graft or infected aortic aneurysm, extensive debridement, and in situ replacement with a Dacron graft, rifampicin-soaked Dacron graft, or cryopreserved homograft is the standard procedure for treating aortic graft infections and aortic infections [9–11]. However, which graft material is most suitable for replacing an infected aorta is a matter of controversy. Although cryopreserved arterial homografts are excellent material for treat infected aortas, the supply in Japan is inadequate, and it is difficult to obtain one in time for an urgent operation. Autologous pericardium has been widely used to treat infective endocarditis, but its surface area is not great enough to reconstruct the great vessels, and bovine pericardium is great enough and twice as stiff as human paricardium [12]. Also, preserving the autogenous pericardium is important to provide a barrier to the infection. Xenopericardium has been widely used to correct congenital cardiac defects, including as atrial and ventricular baffles and as a patches or valved conduits to enlarge the right ventricular outflow tract and main pulmonary artery. However, few reports describe using xenopericardium to construct a neo-aorta. Bovine pericardium preserved in glutaraldehyde began to be used to enlarge the ascending aorta in 1979, and valved tubes were later used for total reconstruction of the ascending aorta [13, 14]. Salles et al. reported their initial early clinical experience with crimped bovine pericardial conduits in ten patients with an aortic dissection or aortic aneurysm [15, 16]. They reported a mean follow-up period of 8 months and that there were no late postoperative complications related to the conduit. They listed the following as positive aspects of this bioprosthetic material: the softness of the biological tissue, which allows easy surgical handling, the good coaptation to suture lines resulting in a hemostatic anastomosis, and the lower thrombogenicity of glutaraldehyde-treated collagen tissue. Crimped bovine pericardial conduits have longitudinal elasticity, maintain their shape with bending, and do not kink even if bent to 180°, however, a specially designed cylinder is required to wrap the pericardial sheet to create the crimps. None of the roll grafts kinked in any of our cases, even when used for aortic arch replacement. Although Salles et al. used the conduits to reconstruct non-infected aortas, it is noteworthy that they mentioned that storage in bacteriostatic glutaraldehyde solution might provide long-term protection against graft infection. A lower incidence of infection in non-crimped bovine pericardial conduits used for replacement of the ascending aorta and arch has been reported in comparison with Dacron grafts [17], and the lower incidence may have been related to the antimicrobial activity of the glutaraldehyde absorbed into the pericardial tissue. Residual glutaraldehyde maintains its antimicrobial properties during surgery and for several hours following conduit implantation [18, 19].

Yamamoto et al. used equine pericardium in a locally-infected field when repairing a ruptured infected abdominal aortic aneurysm and confirmed excellent durability of the graft and absence of graft infection during long-term follow-up [20]. They also reported a case of successful in situ replacement of the thoracic descending aorta with an equine pericardial roll graft and left lower pulmonary lobectomy for an aortobronchial fistula secondary to aortic rupture caused by infection by α–streptococci [21]. Omentopexy was not performed in that patient, because omental mobilization was considered impossible due to a past history of laparotomy for an esophageal hiatal hernia. There were reasons for avoiding omentopexy in our own cases: a history of open abdominal surgery, the presence of an abdominal infection site, and hemodynamic instability. In cases that require a second stage esophageal reconstruction with a gastric roll, it is better to avoid gastrointestinal procedures, including omentum harvesting. Occasionally the lesion in cases of aortic arch reconstruction is too distant to wrap the graft and its branches with the omentum. While pedicled muscle flaps are an alternative means of protecting grafts, they are less effective than omental flaps.

Czerny et al. reported the results of using bovine pericardial tube grafts to treat prosthetic graft infection or endovascular graft infection in 15 patients [22]. They concluded that treating postoperative graft infections or performing endovascular treatment of thoracic, thoracoabdominal, and abdominal aortic diseases by complete removal of the infected prosthetic material and extensive debridement followed by orthotopic vascular reconstruction with intraoperatively prepared xenopericardial tube grafts as neoaortic segments provides excellent results with regard to durability and freedom from reinfection and reoperation. They also mentioned that this new concept: bovine pericardial tube grafts may be superior to cryopreserved homografts because the likelihood of calcification seems to be less important and that another advantage of customized xenopericardial tissue is its availability, which turns out to be a problem with homografts. *Line break* Pericardial sheets are soft and easy to handle. They can be formed into cylinders intraoperatively by rolling them up, and they provided us with a good operative field. It was easy to adjust the diameter of the roll graft to the diameter of the transected aorta. 10 cm of side length of the pericardium is ideal for construction of a neo-aorta without trimming. When we make a 30 mm diameter of the graft, a margin length to sew up was calculated as (10–3 π) / 2 = 0.3 cm. Graft dilatation, mural thrombus formation, shrinkage, calcification, and recurrence of the infection are concerns during long-term follow up. Contrast-enhanced computed tomography may be the most suitable imaging examination for follow-up. None of the above complications occurred in any of our cases. Two patients required TEVAR or direct closure to treat pseudoaneurysms of the anastomoses and to treat pseudoaneurysms of the suture line between the two edges of the pericardial sheet. Based on a comparison of the intraoperative photographs and computed tomography findings, the main cause of the pseudoaneurysms appeared to be a coarse continuous suture that had loosened. Although it is unclear why these pseudoaneurysms did not develop soon after the operation, we think that closer sutures may prevent the occurrence of the pseudoaneurysms in the long-term.

The pathogen and graft material also affect the prognosis. The case of a patient who died of septic shock in the early postoperative period in which histological examination revealed MRSA colonization and a damaged inner layer of the equine pericardial roll graft has been reported [23]. The authors of the case report suggested that the barrier function of equine pericardium against bacterial colonization may be inadequate in patients with MRSA sepsis and result in structural instability due to tissue destruction. Degeneration of the conduit wall and subsequent development of a pseudoaneurysm must result from an acute inflammatory reaction and the presence of a large number of macrophages and intense phagocystic activity by inflammatory cells, including polymorphonuclear leukocytes, activated macrophages, and neutrophils releasing protective enzymes. This in turn may induce structural degeneration of collagen and result in fragmentation as well as the release of proteolytic enzymes such as collagenase and elastase by microorganisms. One of our MRSA infection patients in whom antibiotic administration was discontinued is alive 13 months postoperatively without any complications. However, the duration of postoperative antibiotic therapy is still a matter of controversy. We routinely prescribe antibiotics according to the Japanese guidelines for the treatment of infectious ‘native valve’ endocarditis because the xenopericardium is a biomaterial. No pathogens were detected in two of our patients, perhaps because of the long preoperative period of intravenous antibiotic therapy.

McMillan et al. used bovine pericardial patches to repair infected fields of 51 peripheral arteries that required removal of infected polytetrafluoroethylene grafts, and although MRSA accounted for 22 % of all organisms detected, 50 of the 51 patches remained in place during a mean follow-up period of 2.1 years without evidence of recurrent infection, rupture, or revision [24]. They concluded that bovine pericardial patch provides a durable alternative to saphenous vein for arterial reconstruction following removal of infected arterial grafts regardless the organism.

Neck vessel reconstruction with xenopericardial branches in our patient has been challenging. A search of the literature retrieved no publications that described the same procedure. A case of aortic arch replacement with a crimped bovine pericardial graft has been reported, but the arch vessels were anastomosed as an “island” [16]. In view of the importance of adequate wide resection of the segment of aorta that contains the infected lesion, the arch vessels in our cases were reconstructed separately. Major concerns are stenosis due to shrinkage of the pericardium and stroke secondary to mural thrombi. Ho et al. described the intermediate-term outcome of carotid endarterectomy with bovine pericardial patch closure in comparison with Dacron patch closure and primary closure [25]. Although they used antiplatelet agents and Coumadin in only 13 and 0.2 %, respectively, of their patients, stenosis had occurred in only 1.1 % of the bovine pericardial patch angioplasty cases at 5 years, and the 5-year survival rate was significantly higher in the group who underwent bovine pericardial patch angioplasty than in the group who underwent Dacron patch angioplasty or primary closure. They concluded that bovine pericardial patch angioplasty following carotid endarterectomy was the best treatment. The need for treatment with anticoagulant or antiplatelet agents is also a matter of controversy. We recommend that patients who have undergone surgical individual reconstruction of aortic arch vessels be treated with an anticoagulant or antiplatelet drug to prevent strokes and graft stenosis secondary to mural thrombi.

In addition to the graft materials commonly used thus far, bioengineered tissue produced from pluripotent or multipotent stem cells, so-called tissue-engineered vascular graft (TEVG) tissue, may provide ideal vascular conduits [26].

The most important advantage of tissue-engineered implants is that the tissue can grow, remodel, rebuild, and respond to injury. According to the first U.S. Food and Drug Administration-approved human clinical trial in the United States of TEVG use in children with congenital heart defects, although asymptomatic graft stenosis developed in the patients with small diameter (<18-mm) conduits, there were no reported thromboembolic, hemorrhagic, or infectious complications, and there was significant growth of the TEVG. Application of TEVG to the creation of neo-aorta is a future task. Accumulation of clinical cases and confirmation of the long-term durability of xenopericardial branched grafts may demonstrate their advantages as an option for the treatment for infected aortic aneurysms.

## Conclusions

Xenopericardial roll graft replacement is simple and less invasive than the standard procedure. Accumulation of clinical cases and confirmation of the long-term durability of xenopericardial branched grafts may show that this procedure has the potential to serve as an option for the treatment of aortic infections and aortic/graft infections as an “initial” treatment as well as a “rescue” treatment.
